# Which patients need anterior cruciate ligament reconstruction after initial treatment with rehabilitation? A scoping review

**DOI:** 10.1002/ksa.12378

**Published:** 2024-07-24

**Authors:** Kamilla Arp, Jacob Nedermark, Kim Gordon Ingwersen, Eva Ageberg, Claus Varnum, Bjarke Viberg

**Affiliations:** ^1^ Department of Orthopedic Surgery Lillebaelt Hospital—Vejle, University Hospital of Southern Denmark Vejle Denmark; ^2^ Department of Regional Health Research University of Southern Denmark Odense Denmark; ^3^ Department of Physiotherapy Lillebaelt Hospital—Vejle, University Hospital of Southern Denmark Vejle Denmark; ^4^ Department of Health Sciences, Faculty of Medicine Lund University Lund Sweden; ^5^ Department of Orthopaedic Surgery and Traumatology Odense University Hospital Odense Denmark

**Keywords:** anterior cruciate ligament, anterior cruciate ligament reconstruction, nonsurgical treatment, predictors, rehabilitation

## Abstract

**Purpose:**

Some patients with anterior cruciate ligament (ACL) injury initially treated with rehabilitation need ACL reconstruction (ACLR); yet, it is unclear what characterizes these patients. This review aimed to describe predictors for ACLR in patients initially treated with rehabilitation.

**Methods:**

A systematic literature search was performed in the Cochrane, Embase, Medline, SportsDiscus and Web of Science databases from inception to 21 February 2023. Articles describing characteristics in adult patients with ACL injury undergoing ACLR after a minimum of 5 weeks rehabilitation were included. It was a priori chosen that characteristics described in at least three articles were considered more certain and could be defined as a predictor for ACLR, and those described in less than three articles were considered less certain and therefore defined as possible predictors. Articles were screened by two independent reviewers. The study was originally intended as a systematic review with meta‐analysis, but in case of limited data, we would convert it to a scoping review, as was the case for this review.

**Results:**

There were 22,836 studies identified, and 181 full texts were screened, of which 10 papers were finally included. Only lower age and higher preinjury activity level were identified as predictors for ACLR. Another 12 possible predictors were identified in single studies. Through an iterative process, potential predictors were categorized into four groups: patient demographics, knee function, patient‐reported outcome measures and anatomical structures.

**Conclusion:**

Lower age and higher preinjury activity level were the only predictors for ACLR after initial treatment with rehabilitation. While younger and highly active patients show a higher need for ACLR, more studies focussing on predictors and reasons for delayed ACLR are warranted.

**Level of Evidence:**

Level II.

**Abbreviations**
ACL: anterior cruciate ligament
ACLR: anterior cruciate ligament reconstruction
BMI: body mass index
GRS: Global Rating Scale
IKDC: International Knee Documentation Committee
PECO: Population, Exposure, Comparator, Outcome
PROMs: patient‐reported outcome measures
QoL: quality of life
RCT: randomized controlled trial

## INTRODUCTION

Anterior cruciate ligament (ACL) injuries are treated with rehabilitation with or without ACL reconstruction (ACLR). Previous research compared functional and clinical outcomes between early ACLR and initial rehabilitation with optional delayed ACLR [2, 20, 35]. It remains unclear if one treatment strategy is superior [37], but studies show that patients treated with rehabilitation alone can achieve similar functional, radiographic and patient‐reported outcomes compared to patients treated with ACLR [19, 20, 54]. Rehabilitation after ACL injury can improve functional stability of the knee and give patients the opportunity to cope with the injury [24] and possibly return to their preinjury sports without ACLR [33, 38]. Studies report that patients can achieve better postoperative outcomes and return to sport when following a period of rehabilitation before ACLR compared to standard treatment [15, 48]. Therefore, a period of rehabilitation before deciding if ACLR is necessary can be recommended [37] and has been adopted in some countries [1, 29, 43]. However, some patients will fail rehabilitation and need delayed ACLR [2, 20, 35]. If ACLR is delayed for too long, it can refrain patients from participating in sports and activities for a longer period. Increased time from injury has been associated with injuries in the medial compartment, for example, medial meniscus injuries [39, 40], suggesting that postponing an ACLR could possibly expose the patient to increased risk of new injuries. Moreover, delaying an ACLR is also suggested to be cost ineffective due to longer sick leave [10, 52].

Guidelines recommend ACLR if patients experience persistent instability despite a period of nonsurgical treatment [11, 29, 43]. Persistent instability (e.g., episodes of giving way or instability of other origin such as symptomatic meniscus tear or loose body giving jack knifing phenomenon) was also used as per protocol criteria to recommend ACLR in patients allocated to rehabilitation with optional delayed ACLR in three randomized controlled trials (RCTs) [2, 20, 35]. Nevertheless, while rehabilitation with optional delayed ACLR is common in clinical practice, it remains uncertain what factors influence the need of delayed ACLR. While previous reviews have compared outcomes following rehabilitation alone or with ACLR, as well as outcomes following early versus late ACLR [4, 31, 37] to our knowledge, no studies have systematically described predictors for ACLR after initial treatment with rehabilitation. A description of what predicts the need for ACLR may contribute to support the clinicians in their evaluation of these patients for a better and more timely treatment for patients with ACL injury to decide whether surgery is needed or not. Therefore, the objective of this scoping review was to systematically identify predictors for patients to undergo ACLR after initially treated with a period of rehabilitation.

## MATERIALS AND METHODS

This review was originally intended as a systematic review with meta‐analysis, but in case of limited data, a conversion to a scoping review would be chosen for better utility of the presentation and discussion of the results. Therefore, we completed this study as a scoping review to use the data, and lack hereof, to provide insights into what is actually known and describe the knowledge gap [34]. The guidelines from Joanna Briggs Institute on scoping review methodology were followed [34].

Reporting was performed according to the Preferred Reporting Items for Systematic reviews and Meta‐Analyses extension for Scoping Reviews guideline [46].

### Eligibility criteria

All original scientific articles available in English were eligible if (1) they included patients with a primary, unilateral ACL rupture following nonsurgical treatment for a minimum of 5 weeks, (2) the majority of included patients were adults with closed epiphyseal plates, (3) a minimum of 10 patients underwent ACLR, (4) ACLR was performed within 1 year from injury, (5) authors described at least one factor associated with ACLR and (6) the study was peer reviewed and available in full text. Studies excluded were those including a majority of paediatric patients (not closed epiphyseal plates), partial ACL ruptures, ACL sutures, cadaveric or animal studies, papers not available in English or not original studies (e.g., reviews, clinical guidelines).

### Definition of predictors

Predictors were defined as factors or characteristics in patients who had delayed ACLR after primarily treated with rehabilitation and were significantly different from patients treated with rehabilitation without ACLR. Predictors were considered to be patient related (i.e., demographics) or related to objective factors (i.e., muscle function, patient‐reported outcome measures [PROMs]). The term ‘predictors’ was used for characteristics described in at least three studies, and ‘possible predictors’ was used for the characteristics described in less than three studies. The predefined limit of at least three articles was based on the original plan to make a meta‐analysis where less than three articles describing a predictor was considered to be less certain. In this paper, the term ‘perceived instability’ will be used to refer to ‘instability’, ‘functional instability’, ‘subjective instability’ and ‘giving way’ which are inconsistently reported in the literature.

### Information sources and search strategy

The search string was based on the PECO criteria:

P: Adults with ACL rupture

E: Nonsurgical treatment with rehabilitation

C: Patients not undergoing ACLR

O: Patients who underwent ACLR

A research librarian assisted developing the search string and adaptation to each database. In that process, comparator was omitted since the group was already covered by the exposure, thus leaving three individual blocks for the search string. Both medical subject headings and free text words combined with Boolean operators and truncations were used when relevant. Five databases (MEDLINE via OVID, EMBASE via OVID, The Cochrane Library, SportDiscus via ESBCO and Web of science) were searched from inception to 21 December 2021 and updated to 21 February 2023 with no language restrictions.

A manual search of reference lists from eligible papers was also conducted to identify further potential studies. An example of the search strategy can be found in File S1.

### Selection of sources of evidence

All references were imported to Covidence where duplicates were removed. Two reviewers (K. A., J. N.) independently screened titles and abstracts for potential eligible studies. These were obtained for full texts and subsequently reviewed by both reviewers to determine the eligibility and included in the final review. Disagreements were resolved by discussion between the two reviewers, and if consensus was not reached, the senior author (B. V.) was consulted.

### Data charting process and data items

Two data chartering forms were developed and tested in the author team before final data extraction. The first form included the following data items: author, publication year, country, study design, number of participants, number of patients undergoing delayed ACLR, duration of rehabilitation and mean age of participants. The second form included all the described possible predictors from each study.

Authors of eligible studies [2, 17, 18, 20, 23, 24, 26, 27, 42] were contacted by email with request for missing or additional data such as the number of failures in a rehabilitation group or possible predictors on outcomes collected but not reported by treatment groups. One of the nine authors provided the requested data [20], five authors were not able to provide additional information [2, 23, 24, 26, 42] and three did not reply [17, 18, 27].

### Critical appraisal of individual sources of evidence

A formal assessment of study quality was not performed in this scoping review.

### Synthesis of results

Possible predictors were extracted from the papers and presented descriptively. These were described as patient‐related or objective factors and were defined through an iterative process. Patient‐related factors included possible predictors related to patient demographics, and objective factors covered possible predictors related to (1) functionally assessed knee function, (2) PROMs and (3) anatomical structures. No between group analyses were made.

## RESULTS

### Study selection

**Figure 1:**
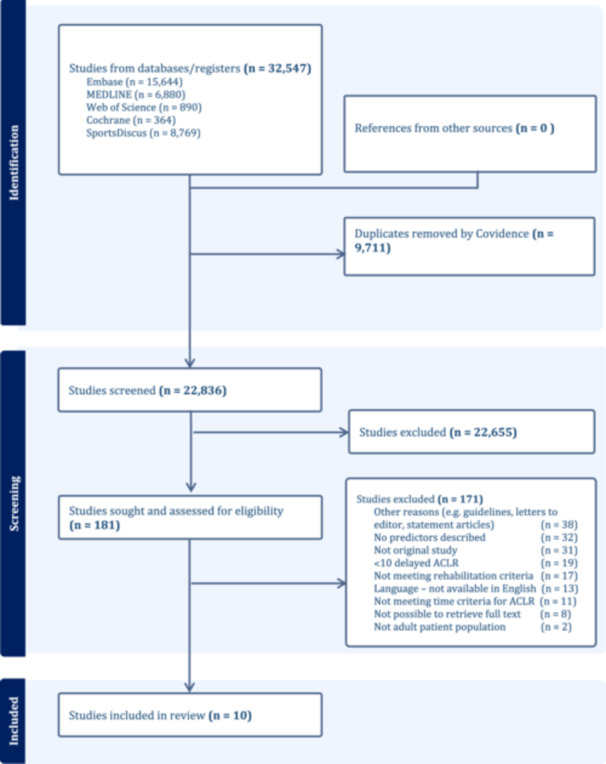
PRISMA‐ScR flowchart of the article selection process. Predictors for delayed ACLR. ACLR, anterior cruciate ligament reconstruction; PRISMA‐ScR, Preferred Reporting Items for Systematic reviews and Meta‐Analyses extension for Scoping Reviews.

The search identified 22,863 studies, of which 181 studies were retrieved for full‐text screening after screening of title/abstract. Of these, 10 studies were included (Figure 1).

### Study characteristics

Two studies were RCTs [20, 49] and eight were prospective cohort studies [13, 14, 16, 24, 30, 32, 44, 51] (Table 1). A total of 1166 patients were included, of which 590 had delayed ACLR. The mean age in studies ranged from 24 to 31 years.

**Table 1:** General characteristics of the included studies.

First author					Age^a^, ^b^		
Publication year							
Country	Study design	Participants primary treated with rehabilitation, *n*, (% female)	Participants undergoing delayed ACLR, *n*	Duration of rehabilitation	Total population	Delayed ACLR	Rehabilitation only
Eitzen [13] 2010 Norway	Prospective	65 (63)	40	4‐6 weeks		23.8 (6.7)^b^	28.6 (8.5)^b^
Eitzen [14] 2010 Norway	Prospective	100 (44)	64	5 weeks	26.1 (14–47)^a^	24.5^a^	29.0^a^
Filbay [16] 2022 Sweden	Prospective	275 (52)	102	Minimum 3 months	25 (7)^b^		
Frobell [20] 2010 Sweden	RCT	59	13	Minimum 5 months		25.1 (23.2–27.0)^a^	26.8 (24.5–27.8)^a^
Grindem [24] 2014 Norway	Prospective	143 (55)	100	Minimum 5 weeks		24.0 (7.2)^b^	30.2 (8.8)^b^
Moksnes [30] 2009 Norway	Prospective	125 (55)	50	Minimum 3 months	27.2 (8.6)	25.9 (8.2)^a^	30.0 (9.1)^a^
Park [32] 2020 South Korea	Prospective	37 (10)	23	Minimum 6 weeks		29.7 (15–49)^a^	33.2 (16–48)^a^
Swirtun [44] 2006 Sweden	Prospective	74 (47)	16	Minimum 5 months	33 (7.8)^b^		
Van der Graff [49] 2022 The Netherlands	RCT	82 (37)	41	Minimum 3 months		27.4 (8.7)^b^	35.3 (11.2)^b^
Van der List [51] 2021 The Netherlands	Retrospective	206 (47)	141	Minimum 6 weeks	31.2 (13.6)^b^	27.4 (11.7)^b^	39.6 (13.6)^b^

Abbreviations: ACLR, anterior cruciate ligament reconstruction; RCT, randomized controlled trial.

^a^ Age reported as median (range).

^b^ Age reported as mean (SD).

### Descriptive data for predictors

**Figure 2:**
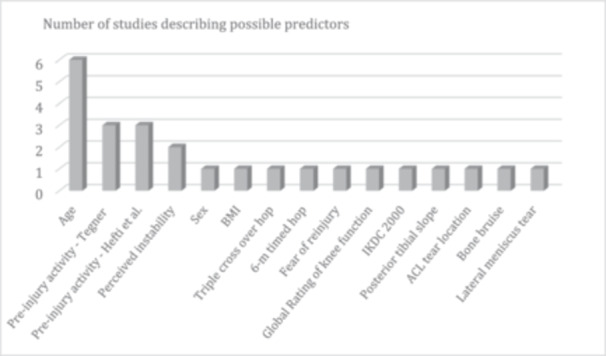
Number of studies describing possible predictors. ACL, anterior cruciate ligament; BMI, body mass index; IKDC, The International Knee Documentation Committee Subjective Knee Evaluation Form.

A total of 14 different possible predictors were identified, whereof two predictors were provided in at least three studies (Table 2, Figure 2). Lower age was identified as a predictor for delayed ACLR in six studies [13, 14, 24, 30, 49, 51], where the mean age ranged from 23 to 27 years for patients having ACLR compared to a mean age from 28 to 39 years in patients without ACLR. Higher preinjury activity level was identified as a predictor in six studies; three studies assessed preinjury activity using the Tegner activity score [44, 49, 51], and three studies [13, 14, 24] used activity level in accordance with Hefti et al. [25]. They found significantly more patients with a Tegner activity level > 7 or participation in level I sports required ACLR*.* Two of the included studies [13, 14] recruited patients from the same clinic with a partial overlap in the recruitment period in these two studies. Despite this, it was decided to include both studies in this review.

**Table 2:** Possible predictors in the included studies.^a^

First author	Possible predictors			
Publication year	Patient related factors	Objective factors		
Country	Patient demographics	Functionally assessed knee function	PROMs	Anatomical structures
Eitzen [13] 2010 Norway	**AGE** **PREINJURY ACTIVITY LEVEL (Hefti et al.)** **SEX** Episodes of giving way	Knee extension strength Knee flexion strength Single leg hop tests: Single hop Triple hop Cross over hop 6‐m timed hop	KOS‐ADLS IKDC‐2000 Global rating of knee function	Difference in anterior laxity
Eitzen [14] 2010 Norway	**AGE** **PREINJURY ACTIVITY LEVEL (Hefti et al.)** Sex BMI Episodes of giving way	Knee extension strength Knee flexion strength Single leg hop tests: Single hop Triple hop Cross over hop 6‐m timed hop	KOS‐ADLS IKDC‐2000 Global rating of knee function	Difference in anterior laxity
Filbay [16] 2021 Sweden	Age Sex Preinjury activity level (Tegner)		Fear of reinjury **ACL‐QoL ITEM 31** ACL‐RSI item 9 K‐SES item D2 General self‐efficacy Knee function (SANE score) Expectation for recovery	
Frobell [20] 2010 Sweden	Age Sex BMI Sport at injury Preinjury activity level **SUBJECTIVE INSTABILITY**		KOOS SF‐36	Baseline meniscal tear Baseline osteochondral injury New/worsening mescal tear
Grindem [24] 2014 Norway	**AGE** Sex BMI **PREINJURY ACTIVITY LEVEL (Hefti et al.)**	Knee extension strength Knee flexion strength	IKDC‐2000	Concomitant injuries Medial meniscus Lateral meniscus
Moksnes [30] 2009 Norway	**AGE** **GIVING WAY** Sex Difference in anterior laxity	Single leg hop tests: Single hop Triple hop **TRIPLE CROSS OVER HOP** **6‐M TIMED HOP**	KOS‐ADLS **GLOBAL RATING OF KNEE FUNCTION** **IKDC‐2000**	
Park [32] 2020 South Korea	Age Sex BMI Time from injury to diagnosis			**POSTERIOR TIBIAL SLOPE** Concomitant injuries
Swirtun [44] 2006 Sweden	**PREINJURY ACTIVITY LEVEL (Tegner)** Difference in anterior laxity		KOOS	
Van der Graff [49] 2022 Netherlands	A**ge** Sex **PREINJURY ACTIVITY LEVEL (Tegner)** **BMI** Time from injury to presentation		IKDC Lysholm Pain (NRS)	Meniscal tear (any) Medial meniscus Lateral meniscus Both Chondral damage MCL Difference in anterior laxity
Van der List [51] 2021 Netherlands	**AGE** **PREINJURY ACTIVITY LEVEL (Tegner)** Sex Time from injury to presentation			Posterior tibial slope **ACL TEARP LOCATION** **PRESENCE OF BONE BRUISE** ALL injury Meniscus tear (any) Medial meniscus **LATERAL MENISCUS** Both

Abbreviations: ACL, anterior cruciate ligament; ACL‐QoL, the Anterior Cruciate Ligament Quality of Life Questionnaire; ACL‐RSI, the anterior cruciate ligament‐return to sport after injury; ALL, anterolateral ligament; BMI, body mass index; IKDC, The International Knee Documentation Committee Subjective Knee Evaluation Form; KOOS, knee injury and osteoarthritis outcome score; KOS‐ADLS, knee outcome score—activities of daily living scale; K‐SES, Knee Self‐Efficacy Scale; MCL, medial collateral ligament; NRS, Numeric Rang Scale; PROM, patient‐reported outcome measures; SANE score, the single assessment numeric evaluation score; SF‐36, short form 36.

^a^ **Bold text in CAPITAL LETTERS** indicates a potential predictors in the individual studies.

There were 12 possible predictors described in less than 3 studies. Three possible predictors were patient‐related factors: sex, body mass index (BMI) and perceived instability. Nine were objective factors, which were categorized into three groups through an iterative process: knee function (triple cross over hop for distance, 6‐m timed hop), PROMs (the Anterior Cruciate Ligament Quality of Life Questionnaire [ACL‐QoL] item 31 [fear of reinjury], Global Rating Scale [GRS] of knee function and International Knee Documentation Committee [IKDC]‐2000) and anatomical structures (posterior tibial slope, ACL tear location, presence of bone bruise and lateral meniscus tear). Additional data on all possible predictors can be found in File S2.

## DISCUSSION

The most important finding in this scoping review is that only lower age and higher preinjury activity were predictors for ACLR in patients initially treated with rehabilitation.

A total of 14 possible predictors to identify patients needing delayed ACLR after initially treated with rehabilitation, but only two of these, that is, lower age and higher preinjury activity level, were found in at least three studies and were defined as predictors for delayed ACLR.

### Patient‐related factors

Possible predictors associated with patient‐related factors were lower age, higher activity level, perceived instability, male sex and lower BMI. Lower age and higher preinjury activity level were the only patient‐related predictors for delayed ACLR identified in this review. Recommending ACLR to patients of younger age is supported in the literature and is typically advised as primary treatment [11]. Younger patients are typically more active in knee‐demanding sports and are less likely to adapt their activity level to prevent surgery compared to older patients [51] arguing for a higher need of ACLR. In the current review, we found that in studies where lower age was a predictor for delayed ACLR, patients had a lower mean age (24–27 years) compared to the studies where age was not as a predictor (30–33 years). For example, van der List et al. [50] reported that 94% of patients below 25 years had ACLR, while the corresponding number was 56% in the age group 25–40 years. This could explain why studies including older patients had a more equal distribution of patients with and without delayed ACLR than studies including younger patients. Yet, while ACLR in the past has been performed primarily in younger patients, studies have found that older patients can achieve good outcomes when undergoing ACLR [5, 36]. The indication for ACLR may be less based on chronological age and more on functional limitations, that is, persistent instability and overall functional status [9]. Although the present review described younger age as a predictor for delayed ACLR, clinicians may consider that delayed ACLR can also be needed in older patients (e.g., if they experience persistent perceived instability) who can also achieve good outcomes following ACLR [37].

Higher preinjury activity level was also a predictor for delayed ACLR, assessed using the Tegner activity scale or activity level in accordance with Hefti et al. [25]. The score on the Tegner activity scale ranges from 0 (disability due to knee problems) to 10 (competitive sports on elite level), while activity level in accordance with Hefti et al. [25] is based on what activity the patient can perform without significant knee pain ranging from level I (very strenuous activities, e.g., pivoting sports) to level IV (light activities, e.g., walking). Three studies [13, 14, 24] in the current review described that patients with a Tegner activity scale of ≥7 (competitive level in, e.g., tennis and recreational soccer) had delayed ACLR more often than those with a Tegner activity scale ˂ 7, and three studies found that patients participating in level I sports [25] were significantly more likely to have delayed ACLR compared to those participating in level II sports. These results are in line with a previous prospective study [8] and a review [7] that also discussed preinjury activity was associated with the need for delayed ACLR. Yet, these studies are nearly 30 years old and did not describe the period or type of rehabilitation patients followed nor at what time point during a longer follow‐up patients had delayed ACLR. However, a recent study reported that a patient's wish to return to preinjury sport was one of the most important factors when recommending ACLR [21], which supports the results from this review. It was not reported in any of the included studies how many patients were sports active on elite level and looking into the data; these have likely been a minority. The evidence from this review is therefore limited to non‐elite athletes. Collectively, when evaluating the need for delayed ACLR, clinicians can consider that patients with a higher preinjury activity level will more often undergo delayed ACLR.

Perceived instability was only found as a possible predictor for delayed ACLR in two studies [20, 30]. This is surprising since persistent instability is commonly accepted as a main reason to recommend ACLR [2, 11, 21, 29], and in a survey study, it was reported to be the single most prominent factor affecting the decision for ACLR from clinicians' perspectives [21]. Persistent instability (i.e., repeated episodes with instability or giving way) can result in, for example, new or worsening of meniscal injuries and thereby possibly in a worse outcome for the patient with increased risk of knee osteoarthritis [41]. The studies by Grevnerts et al. [22] and Swirtun et al. [44] did not describe perceived instability as a predictor for ACLR; however, when asking their patients about the reasons for choosing ACLR, perceived instability was the most frequently stated reason. Furthermore, the latter study [44] revealed that the reasons for choosing early ACLR (˂6 months after injury) were based on *assumptions* on future knee demands and possible limitations due to the knee injury, whereas the reasons for choosing delayed ACLR (˃6 months after injury) were based on *experienced* perceived instability and inability to perform preinjury activities. Another study reported that fear of reinjury rather than actual decreased functional ability could be more related to choosing ACLR [24]. Yet, while perceived instability was not a predictor for delayed ACLR, this may be due to the limited number of studies evaluating this factor (i.e., only 4 of the 10 included studies in this review). Perceived instability is a common reason to recommend ACLR, but clinicians can consider if a patient's perceived instability is based on fear and assumptions on future limitations rather than experienced perceived instability despite a period of rehabilitation. The different reasons for choosing ACLR could also indicate that some patients might have a strong treatment preference for surgery which could also be considered a possible predictor for delayed ACLR. This is an area of research that is not well explored. Yet, an exploratory analysis from the COMPARE trial found that, in the group of patients having delayed ACLR, 29% had a strong preference for surgery and of these 75% had an ACLR within 3 months after completing the rehabilitation period [49]. While it can appear that some patients are in a rush to undergo ACLR, patients may have unrealistic high expectations to outcomes following ACLR. Webster and Feller [53] reported that 88% of patients prior to a primary ACLR expected to return to sport on their preinjury level. This expectation is inconsistent with the average return to sport rate as reported in a systematic review [28], where 72% of soccer players returned to play after ACLR but only 53% return to their preinjury level. Clinicians may consider if a patients’ preference for surgery and high expectations to the outcomes can hinder them in the attempt to manage their injury with rehabilitation only.

### Objective factors

Objective factors as possible predictors for delayed ACLR included worse performance on two single legged hop tests (triple cross over hop for distance and 6‐m timed hop), lower ACL QoL item 31, lower GRS of knee function, lower IKDC‐2000, increased posterior tibial slope, proximal ACL tear location, presence of bone bruise and lateral meniscus injury.

Measures of physical performance (i.e., single‐legged hop tests) are frequently used to assess outcomes after ACL injury and ACLR [6, 55] and is used when evaluating the need for delayed ACLR [13, 45]. In the current review, however, only one study found that poorer performance on two single legged hop tests were possible predictors for delayed ACLR [30]. The same study also found that patients choosing delayed ACLR had more episodes of perceived instability and lower IKDC score, which could suggest that patients with worse function early after injury may be more susceptible for delayed ACLR. In the study by Eitzen et al. [13], the authors observed, besides higher preinjury activity level as a predictor for delayed ACLR, that incorporating results from functional performance tests and PROMs into a regression model increased the ability to explain those who had delayed ACLR. Moreover, evaluation of muscle strength and functional performance are key items for patients with ACL injuries [3, 47] and are used to evaluate function during, for example, a rehabilitation process as well as to identify deficiencies in knee function.

An objective measure that was not described as a possible predictor in this review but is often used in the clinical assessment is anterior laxity. Measures of anterior laxity is used to confirm the clinical diagnosis and is an outcome measure after ACLR, where passive stability should be re‐established. As the most obvious objective factor, the degree of anterior laxity has been used to evaluate the need for ACLR. However, the literature is not consistent on this. In the present review, all studies that evaluated baseline anterior laxity found no difference between those who later had ACLR and those who did not [13, 14, 30, 32, 44, 49].

So, although the present scoping review only found functional performance tests and PROMs as possible predictors for delayed ACLR, objective measures are important when evaluating treatment effect and changes after, for example, rehabilitation and may improve the identification of patients needing delayed ACLR and need to be evaluated in future studies. The importance of including a functional perspective in the evaluation of identifying the need for ACLR has been adapted by researchers in proposed screening algorithms. An original screening algorithm was designed to evaluate athletes early after injury regarding who could return to their sport for a period of time without sustaining new injuries (copers) [12]. The algorithm included four aspects, of which one was a single leg hop test (6‐m timed hop) [12]. It has later been shown that the same algorithm might be more accurate to identify who needs delayed ACLR if the screening algorithm is used after a period of 5 weeks of rehabilitation [45]. However, still a large part of patients who were copers after a period of rehabilitation ended up having delayed ACLR [45]. This suggests that while this screening algorithm includes several possible predictors for delayed ACLR, it might not be sufficient to identify the actual need for ACLR or that 5 weeks of rehabilitation may be insufficient time to test the rehabilitation before deciding if ACLR is necessary. Meanwhile, it remains to be evaluated if this algorithm includes the adequate elements or predictors to identify the need for ACLR or if more than 5 weeks of rehabilitation could improve the identification of patients who need delayed ACLR.

### Clinical relevance

Collectively, it seems that both patient‐related and objective factors can be possible predictors for the need of delayed ACLR, but only two predictors were described in a sufficient number of articles: lower age and higher preinjury activity level. Although clinical guidelines and treatment criteria in research recommend ACLR in patients with persistent perceived instability [20, 43], this was not reported in a sufficient number of included articles in this review. However, clinicians can be aware of patients reporting perceived instability and whether this is based on fear and assumptions or on actual experienced perceived instability after rehabilitation.

Since initial rehabilitation after ACL injury is recommended to most patients, at least in some countries, it is important that clinicians can identify when and in which patients delayed ACLR is needed. While it was described that younger patients and those with a higher preinjury activity level more often have delayed ACLR, clinicians must be aware that the current evidence besides these two predictors is very limited.

Further research is needed and should prospectively evaluate patients that follow rehabilitation as first line on treatment and seek to identify which patients need ACLR. Additionally, it is relevant to investigate the patients' perspectives or reasons for choosing delayed ACLR over rehabilitation alone.

### Methodological considerations

Some methodological considerations are present in our scoping review. Only articles describing patients initially treated with rehabilitation for at least 5 weeks and where ACLR was performed within 1 year after injury were included. Predictors from this review do therefore not describe patients who follow a shorter period of initial rehabilitation or if delayed ACLR is considered a longer time since injury. Moreover, the type and quality of rehabilitation may vary between studies as well as the patients' adherence to the rehabilitation protocols, which was not evaluated in the included articles but may influence if a patient needs delayed ACLR. Another consideration is that while some articles [20, 49] described a protocol for recommending delayed ACLR, it must be considered that the choice of delayed ACLR can vary and be based on subjective decisions from both the patient and orthopaedic surgeons. Also, articles were only included if a least 10 patients had delayed ACLR which may exclude other possible predictors in smaller samples. However, this criterion was used to ensure that a characteristic could actually be considered a possible predictor in this group of patients. We did not assess the study quality of the included articles on why the quality of the studies is uncertain. However, when more studies are conducted on this topic, this would be relevant to consider. This study was originally planned as a systematic review with meta‐analysis which was not performed due to very limited available evidence and results must therefore be interpreted with caution.

## CONCLUSION

The evidence regarding which patients need delayed ACL reconstruction is limited. In this scoping review, only two predictors for ACLR after initial treatment with rehabilitation were identified in non‐elite patients, that is, lower age and higher preinjury activity level. Several other possible predictors were also described in the included articles but were considered less certain as they were described in less than three studies. Future prospective studies are necessary and should evaluate the need for ACLR as well as short‐ and long‐term outcomes to following initial rehabilitation.

## AUTHOR CONTRIBUTIONS

The idea for this review was generated by Kamilla Arp, Claus Varnum, Kim Gordon Ingwersen and Bjarke Viberg. Kamilla Arp performed the literature searches, Kamilla Arp and Jacob Nedermark performed the data analysis with support from Bjarke Viberg, Kamilla Arp wrote the first draft of the manuscript and all authors read and provided critical comments and approved the final version of the manuscript.

## CONFLICT OF INTEREST STATEMENT

The authors declare no conflict of interest.

## ETHICS STATEMENT

No ethics approval was required for the present study.

## Supporting information

Supporting information.

Supporting information.

## Data Availability

The data from the present study is available and can be shared upon reasonable request.
