# Supplementary material for: Which patients need anterior cruciate ligament reconstruction after initial treatment with rehabilitation? A scoping review
Source: Knee Surg Sports Traumatol Arthrosc. 2024 Jul 24;33(2):500–12. doi: 10.1002/ksa.12378 (PMC11792103; doi:10.1002/ksa.12378)
Supplement: Supplementary file 1 — Supporting information. [file KSA-33-500-s002.docx]

**Supplementary information 1 Example of the search string from Embase**

**Supplementary information 1** Example of the search string from Embase

**Link:**
[Click to run search](https://proxy1-bib.sdu.dk:2048/login?url=https://ovidsp.ovid.com/ovidweb.cgi?T=JS&NEWS=N&PAGE=main&SHAREDSEARCHID=uvSX9SmxgGgJMBvtx24de4qUW6HPumGf9S3noEHpCUeLCeR99JQuTJbAs5JrowAr)
The above Jumpstart will only work for users who have access to this specific database.


**Database:**
Embase Classic+Embase <1947 to 2021 December 17>

| **#** | **Query** | **Results from 21 Dec 2021** |
| --- | --- | --- |
| 1 | exp anterior cruciate ligament injury/ | 11,198 |
| 2 | exp anterior cruciate ligament/ | 12,733 |
| 3 | exp ligament injury/ | 25,474 |
| 4 | Anterior cruciate ligament.mp. | 31,676 |
| 5 | Ligamentum cruciatum anterius.mp. | 9 |
| 6 | ACL.mp. | 24,863 |
| 7 | Ligament injur*.mp. | 17,538 |
| 8 | Knee injur*.mp. | 21,043 |
| 9 | Joint instab*.mp. | 13,622 |
| 10 | 1 or 2 or 3 or 4 or 5 or 6 or 7 or 8 or 9 | 77,104 |
| 11 | exp conservative treatment/ | 674,271 |
| 12 | exp rehabilitation/ | 455,973 |
| 13 | exp rehabilitation research/ | 1,156 |
| 14 | exp rehabilitation patient/ | 1,525 |
| 15 | exp exercise/ | 405,358 |
| 16 | exp physiotherapy/ | 104,953 |
| 17 | exp physiotherapy practice/ | 1,021 |
| 18 | exp training/ | 97,082 |
| 19 | (non-surg* or nonsurg* or non surg* or non-operat* or nonoperat* or non operat* or conserv*).mp. | 706,489 |
| 20 | ACL insufficien*.mp. | 207 |
| 21 | ACL deficien*.mp. | 1,448 |
| 22 | Rehabilitation.mp. | 424,362 |
| 23 | Exercise.mp. | 575,604 |
| 24 | Training.mp. | 737,890 |
| 25 | Physical ther*.mp. | 44,370 |
| 26 | physiother*.mp. | 136,513 |
| 27 | prehabilitation.mp. | 1,451 |
| 28 | 11 or 12 or 13 or 14 or 15 or 16 or 17 or 18 or 19 or 20 or 21 or 22 or 23 or 24 or 25 or 26 or 27 | 3,027,300 |
| 29 | exp anterior cruciate ligament reconstruction/ | 12,674 |
| 30 | exp allograft/ | 50,576 |
| 31 | exp autograft/ | 17,767 |
| 32 | exp orthopedic surgery/ | 570,223 |
| 33 | Reconstruction.mp. | 366,211 |
| 34 | Allograft*.mp. | 128,637 |
| 35 | Autograft*.mp. | 31,765 |
| 36 | homograft*.mp. | 11,092 |
| 37 | Arthroscop*.mp. | 54,156 |
| 38 | Orthopedic*.mp. | 136,338 |
| 39 | Orthopaedic*.mp. | 73,100 |
| 40 | joint surg*.mp. | 13,513 |
| 41 | Knee surg*.mp. | 8,414 |
| 42 | Rupture surg*.mp. | 315 |
| 43 | Ligament surg*.mp. | 8,237 |
| 44 | 29 or 30 or 31 or 32 or 33 or 34 or 35 or 36 or 37 or 38 or 39 or 40 or 41 or 43 | 1,117,657 |
| 45 | 10 and 28 and 44 | 13,354 |

exp anterior cruciate ligament injury/
exp anterior cruciate ligament/
exp ligament injury/
Anterior cruciate ligament.mp.
Ligamentum cruciatum anterius.mp.
ACL.mp.
Ligament injur*.mp.
Knee injur*.mp.
Joint instab*.mp.
1 or 2 or 3 or 4 or 5 or 6 or 7 or 8 or 9
exp conservative treatment/
exp rehabilitation/
exp rehabilitation research/
exp rehabilitation patient/
exp exercise/
exp physiotherapy/
exp physiotherapy practice/
exp training/
(non-surg* or nonsurg* or non surg* or non-operat* or nonoperat* or non operat* or conserv*).mp.
ACL insufficien*.mp.
ACL deficien*.mp.
Rehabilitation.mp.
Exercise.mp.
Training.mp.
Physical ther*.mp.
physiother*.mp.
prehabilitation.mp.
11 or 12 or 13 or 14 or 15 or 16 or 17 or 18 or 19 or 20 or 21 or 22 or 23 or 24 or 25 or 26 or 27
exp anterior cruciate ligament reconstruction/
exp allograft/
exp autograft/
exp orthopedic surgery/
Reconstruction.mp.
Allograft*.mp.
Autograft*.mp.
homograft*.mp.
Arthroscop*.mp.
Orthopedic*.mp.
Orthopaedic*.mp.
joint surg*.mp.
Knee surg*.mp.
Rupture surg*.mp.
Ligament surg*.mp.
29 or 30 or 31 or 32 or 33 or 34 or 35 or 36 or 37 or 38 or 39 or 40 or 41 or 43
10 and 28 and 44


<https://proxy1-bib.sdu.dk:2048/login?url=https://ovidsp.ovid.com/ovidweb.cgi?T=JS&NEWS=N&PAGE=main&SHAREDSEARCHID=uvSX9SmxgGgJMBvtx24de4qUW6HPumGf9S3noEHpCUeLCeR99JQuTJbAs5JrowAr>
