# Supplementary material for: Which patients need anterior cruciate ligament reconstruction after initial treatment with rehabilitation? A scoping review
Source: Knee Surg Sports Traumatol Arthrosc. 2024 Jul 24;33(2):500–12. doi: 10.1002/ksa.12378 (PMC11792103; doi:10.1002/ksa.12378)
Supplement: Supplementary file 2 — Supporting information. [file KSA-33-500-s001.docx]

**Supplementary information 2 Possible predictors described in the included studies**

| **Supplementary information 2** Possible predictors described in the included studies | | |  |
| --- | --- | --- | --- |
|  | Possible predictor | Delayed ACLR | Rehabilitation only |
| Eitzen et al. (1) | - Age (years) | 23.8 (6.7) *^a^* | 28.6 (8.5) *^a^* |
|  | - Preinjury activity level (Hefti et al), n (%) level I/ level II | 33 (82) / 7 (18) | 9 (36) / 16 (64) |
|  | - Sex, n (%) female | 21 (53) | 20 (80) |
| Eitzen et al. (2) | - Age (years) | 24.5 *^a^* | 29.0 *^a^* |
|  | - Preinjury activity level (Hefti et al), % level I/ level II, (%) | 81 / 19 | 50/50 |
|  |  |  |  |
| Filbay et al. | - Fear of re-injury at 3 months follow up (ACL-QoL-item31) | 27 (21-32) *^b^* | 37 (31-43) *^b^* |
|  |  |  |  |
| Frobell et al. | - Subjective instability, n (%) | 6 (46%) | 11 (23%) |
| Grindem et al. | - Age (years) | 24.0 (7.2) *^a^* | 30.2 (8.8)*^a^* |
|  | - Preinjury activity level (Hefti et al), n (%) level I / level II | 80 (80) / 51 (51) | 19 (44) / 30(70) |
|  |  |  |  |
| Moksnes et al. | - Age (years) - Triple cross hop (% of un-injured) - 6-m timed hop (% of un-injured) - Global rating of knee function (VAS 0-100) - IKDC 2000 - Giving way (number of episodes) | 25.9 (8.2)*^a^*  83.6 (13.2) *^a^*  86.6 (12.1) *^a^*  51.8 (22.5) *^a^*  63.7 (15.2) *^a^*  1 (1-2) *^c^* | 30.0 (9.1) *^a^*  89.7 (11.4) *^a^*  93.0 (10.8) *^a^*  66.0 (19.8) *^a^*  73.4 (10.4) *^a^*  0 (0-1) *^c^* |
|  |  |  |  |
|  |  |  |  |
|  |  |  |  |
| Park et al. | - Posterior tibial slope, mean° (SD) range | 10.2° (2.7°) 5.7°~14.9° | 8.3° (2.4°) 4.3° ~11.9°) |
| Swirtun et al | - Preinjury activity level (Tegner)* | TAS 8-9 | TAS 4-6 |
| Van der Graff et al. | - Age (years) | 27.4 (8.7) ^a^ | 35.3 (11.2) ^a^ |
|  | - Preinjury activity level (Tegner) | 7.6 (1.7) ^a^ | 6.6 (2.1) ^a^ |
|  | - BMI | 24.0 (3.7) ^a^ | 26.0 (3.7) ^a^ |
|  | - ACL injured during sports, n (%) | 32 (78%) | 39 (95.1%) |
| Van der list et al. | - Age (years) - Preinjury activity level (Tegner) - Tegner activity level ˃7, n (%) - ACL tear location (proximal remnant length) - Presence of bone bruise (on lateral femoral condyle), n (%) - Lateral meniscus only, n (%) | 27.4 (11.7)^c^  6.5 (0.9) ^c^  90 (63.8%)  13 ± 4 mm^c^  93 (66%)  23 (16.3%) | 39.6 (13.6) ^c^  5.7 (1.1) ^c^  14 (21.5%)  12 ±3 mm^c^    23 (35.4%)  3 (4.6%) |
|  |  |  |  |
|  |  |  |  |
|  |  |  |  |
|  |  |  |  |

*^a^* Reported as mean (SD or range), *^b^* Estimated marignal mean (95% CI), *^C^* Reported as median (range), *Odds ratio (95%CI) for Tegner level 8-9 vs 4-6 for choosing late reconstruction vs. on-operative, OR = 7.8 (12-52.9)
